# Structural studies of local environments in high-symmetry quasicrystals

**DOI:** 10.1038/s41598-023-42145-7

**Published:** 2023-10-04

**Authors:** Alan Rodrigo Mendoza Sosa, Atahualpa S. Kraemer, Erdal C. Oğuz, Michael Schmiedeberg

**Affiliations:** 1 Departamento de Física, Facultad de Ciencias, Universidad Nacional Autónoma de México, Ciudad Universitaria, 04510 Mexico City, Mexico; 2 CAS Key Laboratory of Soft Matter and Biological Physics, Institute of Physics, Chinese Academy of Sciences, Beijing, 100190 China; 3 Institut für Theoretische Physik, Friedrich-Alexander-Universität Erlangen-Nürnberg, 91058 Erlangen, Germany

**Keywords:** Statistical physics, Computer science, Statistics

## Abstract

The statistics of how the local environment of a particle looks like, e.g., given by the distribution of nearest neighbor distances or the sizes of Voronoi cells, is important as a starting point for the calculation of many material properties like electronic or photonic band structures. Here we study local environments that occur in quasicrystals with large rotational symmetry. Both with analytical considerations based on geometric arguments and with an analysis of a large number of numerically created patches of high-symmetry quasicrystals we find that the Voronoi area’s distribution reaches a bimodal curve and that in the limit of large rotational symmetries the distribution of nearest neighbor distance converges against a universal curve, where $27.7\%$ of the vertices have their nearest neighbor at a normalized distance equal to 1, while for the other $72.3\%$ the nearest neighbor is at a distance less than 1. Therefore, the statistics of local environments is non-trivial but independent of the specific rotational symmetry. Thus properties that only depend on local environments are expected to be universal for all high-symmetry quasicrystals.

## Introduction

The spatial organization of atoms or molecules in a solid-state material ultimately determines its physical and chemical properties. The local environment referring to the arrangement of the constituent elements in the immediate vicinity of a particular atom or site within a material can have a significant impact on various properties of the material, such as its mechanical strength, thermal conductivity, electrical conductivity, and even its optical properties.

Unlike in ordinary crystals, where atoms are arranged in a periodically repeating pattern, the local environments in quasicrystals exhibits a higher degree of complexity; Quasicrystals are aperiodic structures that possess long-ranged order while lacking translational symmetry^1^ and they are characterized by the presence of specific structural motifs (‘tiles’) that are repeated aperiodically throughout the material.

First quasicrystals were observed in an Aluminum-Manganese mixture^1^ about forty years ago, and have been since found in various systems, ranging from metallic alloys (see, e.g.,^2,3^) to intrinsic nanoparticle compounds^4,5^, induced colloidal structures^6–8^, micellar quasicrystals^9^, and beyond to naturally occurring quasicrystalline meteorites^10,11^.

**Figure 1:**
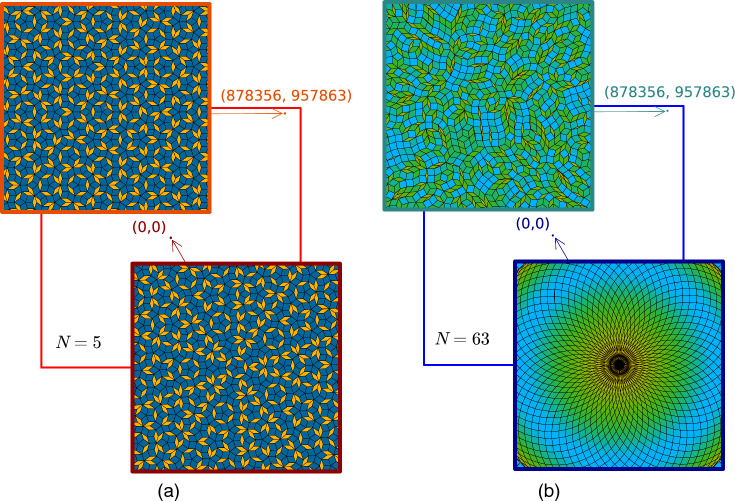
Quasicrystal tilings close and far from the center of symmetry. (**a**) Quasiperiodic tiling of low rotational symmetry $N = 5$ with a zoom around the symmetry center (0, 0), and far from the symmetry center at (878356, 957863). (**b**) Quasiperiodic tiling with a high rotational symmetry $N = 63$ with a zoom around the same two points as in (**a**).

For any given *N* it is possible to generate a quasicrystal with $N-$fold rotational symmetry, e.g., by using the method that we use here. Although this infinite variety in rotational symmetry is considered as one of the most important properties of quasicrystals, only a handful of low-symmetry quasicrystals with $N=5,8,10,12$ have been observed and their local environments are studied in detail^8,12,13^, with rare exceptions like 18-fold symmetry^9,14^. So while intrinsic quasicrystals with large rotational symmetry are rare, in principle they can be induced by external forcing, e.g., by applying laser fields with the desired quasicrystalline symmetry to colloidal systems^8,13^. At a first glance even perfect (i.e., deterministacally calculated) quasicrystalline patterns look similar to random tiling patterns or even other random patterns, at least for large rotational symmetry if only a local patch far from the global symmetry center is shown as in the top panel of Fig. 1(b). This inevitably leaves us with a fundamental question: What are the structural differences, specifically in local environments, between low- and high-symmetry ($N \gg 1$) quasicrystals? And while long-ranged correlations obviously are special for perfect quasicrystals one might ask how a quasicrystal with large rotational symmetry locally differs from random patterns. Could an amorphous structure even be modelled with a well-ordered quasicrystal where all degrees of freedom and excitations in principle are known? Note, that the statistical sampling of high-symmetry quasicrystals requires larger (or more) patches as similar features only repeat at very large distances in contrast to low symmetry quasicrystals where local patterns like local symmetry centers occur more frequently^8,13^.

In this work, we focus on local structural properties of quasicrystals for various *N*. The statistics of local properties like the distribution of nearest neighbor’s distances are essential for the modeling of the interaction energy in stable or metastable structures, the local dynamics like self-diffusivity, the electronic band structures in atomic systems as, e.g., given by the tight-binding model^15^, or the photonic bandstructure in colloidal structures. Note that there are also physical properties that depend on long-ranged correlations, e.g., the multifractal wavefunctions studied for quasicrystals in^16^. However, in this work we will concentrate on the local statistics. Local environments can also affect the stability and formation of quasicrystals: The growth of a quasicrystal often is a process with two steps, namely first a structure with a lot of phasonic strain is assembled and then the strain slowly is relaxed by local rearrangements^17,18^. The structure obtained in the first step differs from the perfect quasicrystal by phasonic flips and therefore locally possess the same statistics concerning nearest neighbor distances. A corresponding statistical analysis is presented in this work. As a consequence, for a grown structure with the same statistical properties as a perfect quasicrystal, one then can determine what phasonic flips are necessary to relax the phasonic strain during the second step of the growth process. Therefore, the statistics of local environments leads to a better understanding of the factors that govern the stability and growth of quasicrystals and is crucial for their controlled synthesis and processing.

In the following, we investigate the pair statistics in two dimensional quasicrystals by means of lattice sites’ next-neighbour distributions with analytical and numerical methods. We further study the Voronoi cell area distributions of these sites using our numerical algorithm. In both cases, we obtain discrete bimodal distributions, where our results suggest that for $N \rightarrow \infty$ these distributions approach a continuous one (albeit dense as dictated by discrete symmetry of the quasicrystal) as the structural complexity increases upon increasing *N*, i.e., a larger number of prototiles are needed to create the lattice.

Our numerical studies require large portions of the quasicrystal. Hence, the main challenge remains in generating large enough and homogeneous high-symmetry quasiperiodic lattices, and far from the perfect symmetry center. We tackle this problem with our improved algorithm which we briefly introduce in the next section alongside our results on structural properties in high-symmetry quasicrystals. Then, we present a discussion on the results obtained and point out some of the lines of research that can be derived from our work. Finally, we conclude our work with some details about the method used to obtain the numerical results.

## Results

Different from periodically ordered materials, quasicrystals can possess arbitrary rotational symmetry^19,20^, allowing an infinite range of possible lattices. As we will see in the following, the previous raises a series of natural questions about the spatial distributions of the vertices of the lattices as a function of the rotational symmetry.

To answer these questions, we investigate local neighborhoods of vertices in a wide range of quasiperiodic lattices, with symmetries ranging from $N=5$ to $N=1009$.

Studying structural properties often requires efficient algorithms to generate the relevant quasiperiodic lattices. We have recently introduced an algorithm^21^ that is based on the Generalized Dual Method (GDM), see Methods section. Our algorithm has successfully reproduced the previous dynamical results published in Ref.^22^. Most importantly, our method circumvents the need for an approximant as it can create perfect quasiperiodic environments at any given point in the space. As such it is also not anymore necessary to limit the studies to a region close to the center of symmetry of the system. Note that, in contrast to low rotational symmetries, for large rotational symmetries the environment around the symmetry center can be very different from the environment further away from it, as illustrated in Fig. 1, where we represent a large quasiperiodic tiling by a red (low rotational symmetry) and blue (high rotational symmetry) square box, and we zoom in around the center of symmetry and far from the center of symmetry. To make plots easier to read, throughout this work, we will use red tones in plots for what corresponds to low rotational symmetries, and blue tones for high rotational symmetries.

### Nearest-neighbour-distance distribution

First, we consider the example of a quasiperiodic tiling with symmetry $N = 5$ formed by the GDM using unitary star vectors. It consists of two types of tiles, (a “fat” and a “skinny” one)^23^. If the side length of the tiles is normalized, then, the tiles can be defined by their minor axis $\bar{a}$, one with $\bar{a} = \sqrt{(5-\sqrt{5})/2}\approx 1.175$ and the other with $\bar{a} = 1/\phi \approx 0.618$, where $\phi$ denotes the number of the golden mean. This yields only two possible nearest-neighbor distances, the unit length 1 and a distance of $1/\phi \approx 0.618$

Increasing the rotational symmetry of the system changes the number of prototiles in a quasiperiodic tiling. As a consequence, the nearest-neighbor distribution also changes. Natural questions are: how many different tiles exist for a given *N*, what is the probability to find each kind of tile, and how does the nearest neighbor distribution change as *N* increases? Furthermore, is there a limit distribution when $N \rightarrow \infty$?

To answer these questions, we use two different approaches, namely analytical and numerical. To be specific, we first analytically calculate the probability of all possible rhombic prototiles for given a symmetry *N*. Then, we approximate the frequency distribution function of the nearest-neighbor distance in the quasiperiodic lattice using this result and assuming that any array of non-overlapping rhombuses around a vertex is possible. The latter is clearly false for low symmetries, where depending on the local isomorphism class some configurations may be prohibited^28^. Despite this, as we will see in the numerical results, for sufficiently high symmetries, this assumption seems to be correct. In our second approach, we compute numerically the nearest-neighbor-distance distribution using our modified GDM algorithm (cf. “Methods” ), and finally we compare our results from both approaches.

### Distribution of tiles

Using GDM we can obtain analytic expressions for the vertices $\vec {t}^{a}_{n_{j}, n_{k}}, a \in [0,1,2,3]$ of the tiles that constitute the quasiperiodic structures^21,24^:

$$\begin{aligned} \vec {t}_{n_{j}, n_{k}}^{\ 0} = n_{j} \vec {e}_{j} + n_{k} \vec {e}_{k} + \sum _{i \ne j \ne k}^{N} \left\lfloor \frac{1}{A_{jk}} \left[ \left( n_{j} + \alpha _{j} \right) \vec {e}_{k}^{\perp } - \left( n_{k} + \alpha _{k} \right) \vec {e}_{j}^{\perp } \right] \cdot \vec {e}_{i} - \alpha _{i} \right\rfloor \vec {e}_{i}, \end{aligned}$$

$$\begin{aligned} \vec {t}_{n_{j}, n_{k}}^{\ 1} = \vec {t}_{n_{j}, n_{k}}^{\ 0} - \vec {e}_{j}, \end{aligned}$$

$$\begin{aligned} \vec {t}_{n_{j}, n_{k}}^{\ 2} = \vec {t}_{n_{j}, n_{k}}^{\ 0} - \vec {e}_{j} - \vec {e}_{k}, \end{aligned}$$

1 $$\begin{aligned} \vec {t}_{n_{j}, n_{k}}^{\ 3} = \vec {t}_{n_{j}, n_{k}}^{\ 0} - \vec {e}_{k}, \end{aligned}$$

where $\vec {e}_k$ are *N* star vectors that define the symmetry of the tiling, $n_k$ are *N* integers associated with each tile, $\alpha _k \in (0,1)$, $A_{jk} = e_{jx} e_{ky} - e_{jy} e_{kx}$ with $e_{kx}$ and $e_{ky}$ the components of the vector $\vec {e}_{k}$, $\vec {e}_{k}^{\perp }$ is the orthogonal vector to $\vec {e}_{k}$, and $\left\lfloor x \right\rfloor$ is the floor function giving the greatest integer less than or equal to *x*. Note that this equation does not require that the star vectors are homogeneously distributed on the circle. However, in this work we will study quasicrystals with rotational symmetry *N*, so the star vectors point to the vertices of a regular polygon with *N* sides. This leads to the fact that if *N* is even, there are *N*/2 star vectors that are antiparallel to the other *N*/2 star vectors. In this case, for each antiparallel pair of vectors $\vec {e}_i$ and $\vec {e}_j$ that fulfill $\alpha _i = n\alpha _j$, Eq. (1) will produce the same result as if choosing only one of the two star vectors. This means that if *N* is of the form $2(2n-1) = 4n-2$, with $n \in \mathbb {N}$ and $\alpha _i = \alpha$ $\forall$ *i*, then Eq. (1) will produce the same tilling as *N*/2, for example, $N = 5$ and $N = 10$ produce the same triangular lattice if all $\alpha _i$ are equal. However, even in this case, if not all the $\alpha _i$ have the same value, then $N = 5$ and $N = 10$ do not produce the same tilling.

By this construction, the sides of the generated tiles have a length of 1 as given by the length of the star vectors that set the unit length. Given this restriction, the tiles only differ from each other by their internal angles. Note that, in our model, the lattice sites correspond to the vertices of the tiles. This implies that the distance *d* between an arbitrary lattice site and its nearest neighbor is bounded from above by the unit length, with $d = \textrm{min}(1, a_{1}, a_{2}, \dots , a_{v})$, *a*’s being the length of the diagonals containing this lattice site (vertex) that is shared by the *v* tiles.

**Figure 2:**
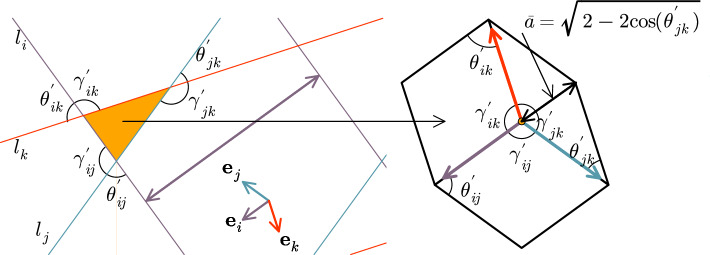
Scheme of the dual transformation. The angles $\gamma _{ij}^\prime$, $\gamma _{ik}^\prime$, $\gamma _{jk}^\prime$, $\theta _{ij}^\prime$, $\theta _{ik}^\prime$ and $\theta _{jk}^\prime$ are the angles form by the intersection of two of the orthogonal lines to the start vectors $\textbf{e}_i$, $\textbf{e}_j$ and $\textbf{e}_k$ forming a triangle area in dual space. They are also the internal angles of the rhombi around a vertex in real space. The small arrows with colors red, blue, and purple represent three of the start vectors orthogonal to the grid lines with the same colors.

The tilings as constructed by the GDM consist of equilateral rhombic tiles with equal opposite angles $\theta ^{\prime }$ and $\gamma ^{\prime } = \pi - \theta ^{\prime }$ in the rhombus with $\theta ^{\prime } \le \pi /2$ being the smaller one. Moreover, each rhombus is produced by a combination of two star vectors $\textbf{e}_j$ and $\textbf{e}_k$ (see Eq. (1) and Fig. 2) with the angles between them corresponding to the two distinct angles in the rhombus. More generally, every pair of star vectors will form an angle of $\theta ^{\prime } = i \pi /N$ with $i \in (2, 4,\dots , N-2)$ for even symmetries and $i \in (2,4,\dots , N-1)$ for odd symmetries. Consequently, the other angle of the rhombus will be $\gamma ^{\prime } = j \pi /N$ with $j \in (N-2, N-4, \dots , 2)$ for even *N* and $j \in (N-2, N-4, \dots , 1)$ for odd *N*.

For even symmetries, because *i* and *j* run over the same values, it is sufficient to consider half of the values of *i*, i.e., there are $\lfloor N/4 \rfloor$ distinct rhombic prototiles. For odd symmetries, however, we need to obtain the angles $\theta = \textrm{min}(\theta ^{\prime }, \gamma ^{\prime })$ for all possible values ($\theta ^{\prime }$ or $\gamma ^{\prime }$), which means half of the values of *i* and half of the values of *j*. Hence there are $\lfloor N/2 \rfloor$ different rhombi. The length of the minor diagonal of a rhombus $\bar{a}$ reads as

2 $$\begin{aligned} \bar{a} = \sqrt{2-2\cos (\theta )}, \end{aligned}$$

where the value of $\bar{a}$ cannot exceed unity, but can well be less than 1 if $\theta ^{\prime } < \pi /3$ or $\gamma ^{\prime } < \pi /3$. In fact, a simple calculation with $\theta ^{\prime }=i\pi /N$ shows that there exists $\lfloor N/6 \rfloor + \lceil \text {mod}(N,3)/N \rceil$ and $\lfloor N/3 \rfloor + \lceil \text {mod}(N,3)/N \rceil$ possible different $\bar{a}$’s (distance to the nearest neighbor) in case *N* is even or odd, respectively.

For $\theta \in [0,\pi /2]$, the values $\bar{a}$ can take according to Eq. (2) are all different. Thus, given a pair of star vectors and integers $n_j$ and $n_k$, producing any rhombus is equally probable, except for the square rhombi ($\theta =\pi /2$) in cases where *N* is a multiple of 4. In these cases the probability to produce a square is half of the probability to produce any other rhombus. In summary, the probability of producing a specific kind of rhombus is $\frac{1}{\lfloor N/2 \rfloor }$ if *N* is odd, $\frac{1}{\lfloor N/4 \rfloor }$ if *N* is even but not a multiple of 4. Moreover the probability is $\frac{4}{(N-2)}$ for non-square rhombi. In case of square rhombi where *N* is a multiple of 4 the probability is $\frac{2}{(N-2)}$. Note that we will not mention explicitly the latter case in the remainder of the manuscript, unless stated otherwise.

Given a random position in real space, we are interested in the probability of finding this position in a particular prototile. This probability is proportional to the area of the prototile since there is an equal probability to construct any type of rhombus. As such, the coverage area of each prototile after producing *n* of them is proportional to the area of each prototile. The area of a rhombus with side length 1 is given by $\sin (\theta )$. Thus, the probability of finding a tile with $\theta$ reads

3 $$\begin{aligned} p(\theta ) = \frac{\sin (\theta )}{\sum _{i=1}^{\lfloor N/2 \rfloor } \sin (2i\pi /N)} = 2\tan \left( \frac{\pi }{2N}\right) \sin (\theta ) \end{aligned}$$

for odd symmetries, and

4 $$\begin{aligned} p(\theta ) = \frac{\sin (\theta )}{\sum _{i=1}^{\lfloor N/4 \rfloor } \sin (2i\pi /N)} = 2\tan \left( \frac{\pi }{N}\right) \sin (\theta ) \end{aligned}$$

for even symmetries that are not multiples of 4.

**Figure 3:**
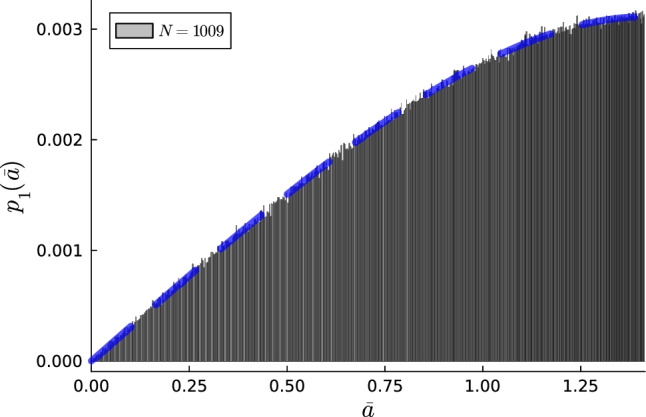
Distribution of rhombic tiles as a function of their minor-diagonal length $\bar{a}$ in a quasiperiodic tiling of symmetry 1009. The histogram was obtained using $1 \times 10^6$ rhombi, and the continuous line is $p_1(\bar{a})$, see Eq. (5).

Alternatively, the probabilities can be expressed in terms of $\bar{a}$; Inverting Eq. (2) yields $\theta (\bar{a}) = \arccos (1-\bar{a}^2/2)$. Thus, the probability of finding a single tile with minor-diagonal length $\bar{a}$ can be written as

5 $$\begin{aligned} p_1(\bar{a}) = \bar{A} \sin \left( \arccos \left( 1-\bar{a}^2/2 \right) \right) , \end{aligned}$$

where $\bar{A} = 1 / \sum _{i=1}^{\lfloor N/2 \rfloor } \sin (2i\pi /N) = 2\tan \left( \frac{\pi }{2N}\right)$ and $\bar{A} = 1 / \sum _{i=1}^{\lfloor N/4 \rfloor } \sin (2i\pi /N) = 2\tan \left( \frac{\pi }{N}\right)$ for odd and even *N*, resp., represents the normalization constant for a rhombus with side length 1. The probability function $p_1(\bar{a})$ in Eq. (5) agrees well with the computationally obtained probabilities, see Fig. 3 for a comparison for $N=1009$.

### Nearest-neighbour-distance

Because each vertex is shared by *v* rhombi in the tiling, the nearest-neighbor distance *d* for this vertex corresponds either to the shortest of the *v* diagonals containing this vertex or to 1 if the shortest diagonal is larger than 1 (note that a vertex can be surrounded by “fat” tiles where both diagonals are larger than 1 and “thin” tiles with the major diagonals passing through the vertex). Then, a first and crude approximation to the probability density of the nearest-neighbor distance would be to assume that there is only one rhombus around a given vertex. In this case, it would be sufficient to consider only a subset of $\bar{a}$’s, namely $a \in [0,1)$ in the probability density to select a diagonal of length *a* (not necessarily the minor one). This is simply given by $\rho _1(a) da = A \sin \left( \arccos \left( 1-a^2/2 \right) \right) \textrm{d}\theta = A a \textrm{d}a$, with $A = 1 / \int _0^2 a'\textrm{d}a' = \frac{1}{2}$

This, of course, is not a good approximation as every vertex belongs to at least three rhombi. Next, we will improve our approximation by considering two rhombic tiles around a vertex. In order to do this, we will assume that any configuration of two rhombi around a vertex is possible in the quasicrystal and then we will estimate the probability that, given a vertex $\textbf{v}$ and a rhombus with a diagonal $a_1$ containing this vertex, there is another rhombus with a diagonal $a_2$ that shares this vertex $\textbf{v}$. We remark that our assumption is not always fulfilled in perfect quasicrystals, but in a random tiling. However, the distribution of rhombi that we use must be equal to that in a perfect quasicrystal. Therefore, we do not expect that the following results hold in more general random tilings. First, we notice that each region (polygon) in the dual space represents a vertex $\textbf{v}$ in the real space, and each vertex in the dual space is a rhombus in the real space (see Fig. 2). Thus, a polygon with *n* vertices in the dual space represents a vertex $\textbf{v}$ in the real space surrounded by *n* rhombi. Fig. 2 shows the transformation of a triangular region in the dual space (left-hand side in Fig. 2) into a vertex in the real space (yellow dot on the right-hand side in Fig. 2), surrounded by 3 rhombi. Because of Eq. (1), the external angles ($\gamma _{ij}^\prime$) of the polygon (triangle) in the dual space are equal to the internal angles of the rhombi around $\textbf{v}$. Moreover, the length of diagonals that pass through $\textbf{v}$ can be calculated using Eq. (2) with $\theta = \theta _{ij}^\prime$, where $\theta _{ij}^\prime$ is the angle formed between the star vectors $\textbf{e}_i$ and $\textbf{e}_j$.

Now consider a segment *s* of a polygon in the dual space formed by the intersection of a grid line $l_i$ orthogonal to $\textbf{e}_i$ with grid lines $l_j$, and $l_k$ orthogonal to $\textbf{e}_j$ and $\textbf{e}_k$ respectively. The polygon is not intersected by any of the grid lines except by those that constitute its boundary as in Fig. 2. Call the intersections between $l_i$ and $l_j$ as $x_{ij}$, and $l_j$ and $l_k$ as $x_{jk}$. If we fix $l_i$ and $l_k$ and displace $l_j$ with respect to $x_{ik}$ in the direction of $\textbf{e}_j$, the length of *s* will change. Since the distance between parallel lines in the dual space is constant and equal to 1, the maximum possible displacement of any line $l_j$ is bounded by 1. In the limit case where there are $N \rightarrow \infty$ star vectors, the maximum length of *s* is 1 because, if not, it would be intersected by another grid line almost orthogonal to *s*. This imposes a maximum displacement $\sin (\theta _{ij}^\prime )$ on $l_j$. Similarly, if we fix $l_i$ and $l_j$ and displace $l_k$, then the maximum displacement for $l_k$ would be $\sin (\theta _{ik}^\prime )$. Then, from all possible displacements $d_j$ and $d_k$ of lines $l_j$ and $l_k$, only those with $d_j <\sin (\theta _{ij}^\prime )$ and $d_k <\sin (\theta _{ik}^\prime )$ can produce the polygon segment with internal angles $\theta _{ij}^\prime$ and $\theta _{ik}^\prime$. Thus, we can approximate the probability to produce a polygon with that segment as $\sin (\theta _{ij}^\prime ) \sin (\theta _{ik}^\prime )$, or equivalently, the probability to have two rhombi around a vertex with angles $\theta _{ij}^\prime$ and $\theta _{ik}^\prime$ is $\sin (\theta _{ij}^\prime ) \sin (\theta _{ik}^\prime ) = \sin \left( \arccos \left( 1-a_1^2 /2\right) \right) \sin \left( \arccos \left( 1-a_2^2 /2\right) \right)$, where we used Eq. (2) to rewrite the probability as a function of the diagonals of the rhombi.

Hence, a second approximation to the probability density of the nearest neighbor distance can be written as:

6 $$\begin{aligned} \rho _2(a) \approx A \sin \left( \arccos \left( \frac{2-a^2}{2}\right) \right) \int _a^{2} \sin \left( \arccos \left( \frac{2-x^2}{2}\right) \right) \textrm{d}x , \end{aligned}$$

with $A = 1/(\int _0^2\int _a^2 \sin (\arcsin (1-a^2 /2)) \sin (\arcsin (1-x^2 /2))\textrm{d}x \textrm{d}a$ being the normalization constant.

Applying the same argument to three or more rhombi is not easy, because, unlike the case of 2, with more rhombi the angles $\theta _1,\theta _2, \dots , \theta _n$ are not all independent, and that is reflected in the fact that three or more randomly chosen rhombi may overlap. However, because $\theta _1 \approx 0$ and $\theta _2 \approx 0$ are very unlikely, we can approximate the probability density function using 3 rhombi as independent. That is:

7 $$\begin{aligned} \rho _3(a) \approx A\sin \left( \arccos \left( \frac{2-a^2}{2}\right) \right) \left[ \int _a^{2} \sin \left( \arccos \left( \frac{2-x^2}{2}\right) \right) \textrm{d}x \right] ^2 , \end{aligned}$$

where $$A = 1/(\int _0^2\sin (\arcsin (1-x^2 /2)) (\int _a^2 \sin (\arcsin (1-a^2 /2)) \textrm{d}a)^2 \textrm{d}x)$$.

Note that for small values of *a*, where one of the rhombi possesses an angle $\theta ^\prime \approx 0$ (thus $\gamma ^{\prime } \approx \pi$, cf. Fig. 2), the probability of the three rhombi overlapping is not so small, such that $\rho _2(a)$ could be a better approximation than $\rho _3(a)$. However, for middle and large values of *a* the 3-rhombi approximation $\rho _3(a)$ should yield more accurate results. In particular, the proportion of nearest neighbors at a distance of 1 should be better approximated with $\int _1^{2} \rho _3(a) \textrm{d}a \approx 0.274$ than with $\int _1^{2} \rho _2(a) \textrm{d}a \approx 0.4223$.

Finally, because the nearest-neighbor distance *d* is given as $d = \textrm{min}(1, a_{1}, a_{2}, \dots , a_{v})$, we can approximate its frequency distribution function using $d \sim \textrm{min}(1, a_{1}, a_{2}, a_{3})$ to yield

8 $$\begin{aligned} \rho (d) \approx {\left\{ \begin{array}{ll} A\sin \left( \arccos \left( \frac{2-d^2}{2}\right) \right) \left[ \int _d^{2} \sin \left( \arccos \left( \frac{2-x^2}{2}\right) \right) \textrm{d}x \right] ^2 &{} \text {if }d < 1,\\ \delta (d-1)\int _1^{2} \rho _3(x) \textrm{d}x &{} \text {if }d \ge 1, \end{array}\right. } \end{aligned}$$

where $\delta (x)$ denotes the Dirac delta function.

At this point, it is worth mentioning that increasing the number of rhombi in our approximation represents a challenging task. First, we would need to calculate the probability $p_n(a)$ of having exactly *n* rhombi around a vertex as a function of *a*, the shortest of all diagonals containing that vertex. Once we would have these probabilities, then we could improve the approximation to $\rho _n(a) = \sum _{i = 1}^n p_i(a) A^i\sin \left( \arccos \left( \frac{2-a^2}{2}\right) \right) \left[ \int _a^{2} \sin \left( \arccos \left( \frac{2-x^2}{2}\right) \right) \textrm{d}x \right] ^{i-1}$ for $n> 3$. This is, however, beyond the scope of this manuscript for two reasons: firstly, there are always at least three rhombi around a vertex, but not necessarily more, and secondly, our numerical measurements show a good agreement with $\rho (d)$ from Eq. (8) as will be shown in the following.

In our previous calculations to obtain $\rho (d)$ we have so far assumed that any configuration of rhombi is possible and that any possible displacement of the lines $l_j$ and $l_k$ is equally probable. As we previously mentioned, strictly speaking, this is incorrect because some configurations can be forbidden in a perfect quasicrystal depending on the local isomorphism class. Therefore, we perform numerical measurements with our algorithm detailed in the Methods section. We generate quasiperiodic lattices and then compute the distribution of the nearest-neighbor distances as a function of the rotational symmetry. Subsequently, we compare our numerical results with the probability density function in Eq. (8). Note that we require a proper sampling for our numerical experiments, i.e. a sufficiently large region and including sufficient parts far away from the symmetry center. For example, the region near the origin in Fig. 1a must not be considered to be representative of the structure at farther distances in general.

**Figure 4:**
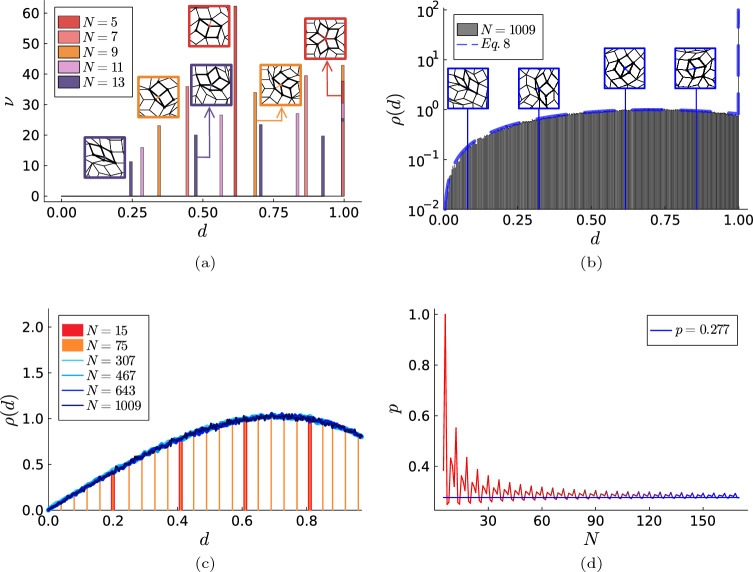
Pair statistics in quasiperiodic lattices. Distribution of the next-neighbor distance of the quasiperiodic lattice sites with $N=5,7,9,11,13$ (**a**), $N = 1009$ (**b**), and $N=15, 75,307,467,643,1009$ (**c**). The dashed line in (**b**) corresponds to our analytical result in Eq. (8). In (**c**) we omit the maximum possible distance $d=1$ plotting with bars for $N = 15$ and $N = 75$ and lines for $N > 75$. (**d**) The probability *p* that the distance to the next neighbor is exactly $d=1$. The blue horizontal line represents the limit value of 0.277 for $N\rightarrow \infty$.

For low symmetries, the distribution of the nearest-neighbor distance exhibits only a few singular peaks at characteristic distances. For instance, for $N=5$, the nearest neighbor of a lattice site is located either at a distance of 1, or at a distance of $1/\tau \approx 0.618$ corresponding to the minor diagonal of the “skinny” tile, cf. Fig. 4a. We embed in the plot some examples of the tiling around a vertex with its nearest neighbor at distance *d*.

For high rotational symmetries, $N \gg 1$, we observe a bimodal distribution of distances containing a singular peak for the distance $d=1$ and a distribution consisting of peaks at all remaining nearest-neighbor distances $d<1$ that are dense in the limit $N\rightarrow \infty$. The limit distribution for $N \rightarrow \infty$ can be approximated by Eq. (8), see the blue dashed line in Fig. 4b. Some examples of the nearest-neighbor distance distribution for $d<1$ are shown in Fig. 4c. While the frequency of the unit distance $d=1$ is larger than other next-neighbour distances $d < 1$, its ratio to distances less than 1 seems to converge to 0.277 as $N \rightarrow \infty$, see Fig. 4d. This agrees fairly well with our analytical expectation $\int _1^{2} \rho (d') \textrm{d}d' \approx 0.274$ using Eq. (8). Figure 4d also shows an oscillatory behavior with period 6 which dampens as *N* grows. We do not understand why there is this period, so it remains an open question. The part with dense peaks for $d<1$ reaches its maximum around a value of $d = \sqrt{2}/2$, corresponding to a tile with a minor angle of $\pi /4$ radians.

### Voronoi area distribution

The distributions obtained in the previous subsection point out three interesting behaviors: (i) It seems that there is no correlation in the type of rhombus around a vertex, (ii) there is a non-constant and well-defined distribution for the next-neighbor distances $d < 1$ of the quasiperiodic lattice sites of high symmetries, and (iii) the ratio of the maximal next-neighbor distance $d=1$ to those where $d < 1$ approximate 0.277. Behavior (i) is unexpected because for low symmetries we know that depending on the class of isomorphism, there are some forbidden configurations of rhombi around a vertex^25^. On the other hand, if the smallest angle of the tiles containing the site exceeds $\pi /3$ radians (“fat tiles”), the nearest neighbor will be at a distance $d = 1$. Thus, $27.7\%$ of all vertices are surrounded by fat tiles or where the major axis of the tile is part of the site. This is relevant because, although growing *N* can produce arbitrarily close neighbors, there will be many more relatively isolated sites. To better understand how isolated each site can be from its neighborhood, we will compute the distribution of the site’s Voronoi cell areas *A* in the following.

**Figure 5:**
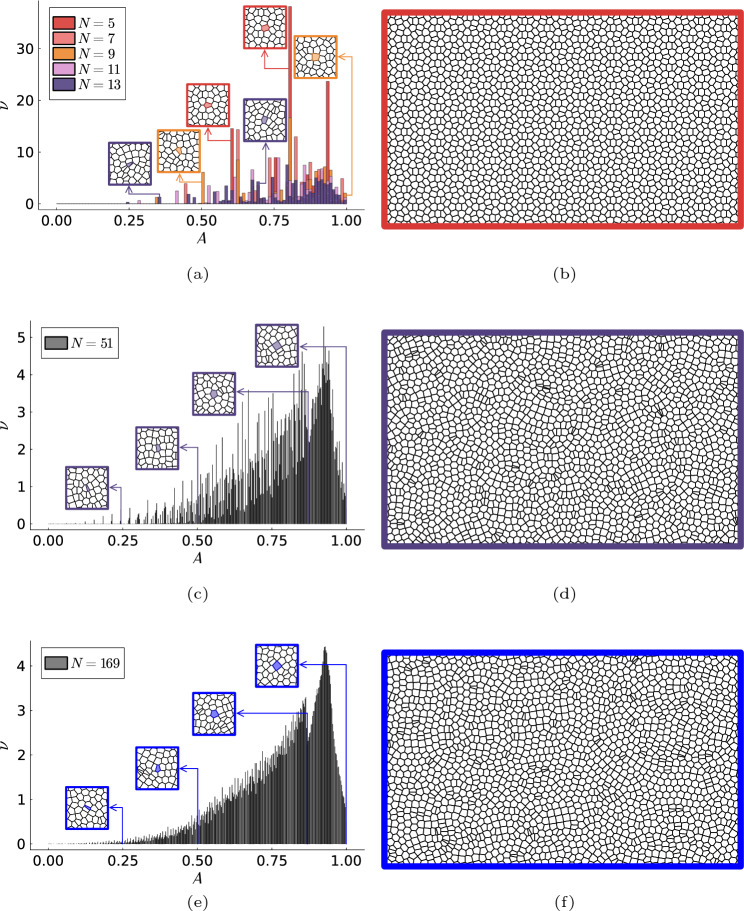
Distribution of the Voronoi areas associated with the sites of the quasiperiodic arrays of rotational symmetry $N=5,7,9,11,13$ (**a**), $N = 51$ (**c**) and $N = 169$ (**e**). Some examples Voronoi tilings for $N=5$ (**b**), $N = 51$ (**d**) and $N = 169$ (**f**).

Similar to the nearest-neighbor distribution, the Voronoi cell area distributions for low rotational symmetries do not show any obvious behavior (see Fig. 5a). However, by increasing the rotational symmetry of the lattices (see Fig. 5c, e), we observe a clear trend towards a well-defined bimodal distribution with a local minimum around the value $A \approx 0.87$ (see Fig. 5e). This is probably related to the fact that there are two kinds of nearest neighbors, those at a distance $d = 1$ and those at a distance $d < 1$. The Voronoi area of those with the nearest neighbor with $d = 1$ is bounded by $A \in (\pi /4, 1]$, where the maximum value corresponds to a vertex surrounded by 4 squares, and the minimum value corresponds to a vertex surrounded by flat rhombi with its smallest diagonal of length 0. In contrast, the Voronoi area of those with $d < 1$ is bounded by $A \in (0, \sqrt{3/4}) \approx (0, 0.87)$, where the minimal value corresponds to a vertex surrounded only by 2 flat rhombi and the maximum value corresponds to a vertex surrounded by 3 rhombi with its smallest diagonal of length 1. Figure 5b, d, f show the Voronoi tesellation for the quasiperiodic lattice sites with rotational symmetry $N=5$, $N=51$ and $N=169$, respectively.

## Discussion

In this work we have introduced and employed an improvement to the algorithm of^21^. This algorithm proved to be helpful in exploring the structural properties of quasicrystals with large rotational symmetries, especially as we now can produce quasicrystalline patterns far away from the global symmetry center. Thus, the statistics can be improved significantly and the results are not distorted by the global symmetry center.

One of our main results is that the distribution of distances to nearest neighbors converges into a universal distribution for $N\rightarrow \infty$ and possesses an unexpected behavior for high symmetries: Approximately a quarter of the particles are exactly one unit length away from their nearest neighbor, while all other particles are closer to their neighbor. Such a distribution cannot be observed for small rotational symmetries. Our result also implies that a perfect quasicrystal with a large rotational symmetry can be used as a model for an amorphous system as long as the same nearest-neighbor-distance distribution and no properties that depend on long-ranged correlations are considered. The advantage of such a model is that the quasicrystals can be obtained deterministically and that in principle all degrees of freedom and all excitations are known. This might help to understand excitations at least in some special amorphous systems.

Our distribution of nearest neighbors can be used as the starting point of tight binding and similar approaches to calculate electronic band structures. Similarly, photonic bandstructures for colloidal quasicrystals might be predicted based on the nearest neighbor distances. Note that there are also physical properties that might depend on long-ranged correlations that we want to study in future works. However, all properties that are mainly based on the local environments interestingly are expected to be similar as the distribution of nearest neighbors converges against the same curve for $N\rightarrow \infty$.

Note that for a given rotational symmetry we can produce different types of quasicrystals with the method that we employ. To be specific, we can continuously tune through different local isomorphism classes^26^. However, we have not observed any changes in our statistical results in the case of large rotational symmetry. Note that our analytical calculations are also independent of the local isomorphism class. In contrast, in quasicrystals with small rotational symmetries, the local isomorphism class might be important, e.g., for photonic properties^28^. This also means that at least for local properties, we can sample quasicrystals with high rotational symmetries by varying the parameters $\alpha _i$ of Eq. 1 homogeneously instead of moving the system far away from the center of symmetry.

All numerical results in this article are obtained for deterministic quasicrystals while for the analytical approximations we considered arbitrary tilings that statistically possess the same composition of tiles around a node as the corresponding deterministic quasicrystals. However, the focus of our work was on the perfect quasicrystals and not on random tilings. Note that for the ensemble of random tilings, an entropy can be defined which was determined in^27^ for high rotational symmetry.

In the future, we want to study the statistical properties on longer length scales. Though the local statistics might be similar for high rotational symmetry, we expect long-ranged correlations that still depend on the rotational symmetry.

## Methods

Our algorithm is based on the Generalized Dual Method (GDM)^19,29^, from which we obtain Eq. (1).

These equations give us a function that, given a set of *N* star vectors, *N* integers, and two indices *j* and *k*, returns a tile of the respective quasiperiodic tiling. However, we are interested in a function that, given a point and the information necessary to produce a given tessellation (the set of star vectors and the displacements associated with the isomorphism class), builds the tessellation around that point. Thus, it would be desirable to obtain the indices *j* and *k*, and the values of the integers $n_{j}$ and $n_{k}$ to produce the tile containing an arbitrary point $\vec {P}$. One possibility is to use brute force, i.e., using all combinations of the indices *j*, *k* and the values for $n_{j}$ and $n_{k}$, starting with 0 and then interleaving *n* and $-n$ beginning with $n = 1$ to find the set of values $n_{j},n_{k}$ that produce the tile. The computational complexity of this algorithm depends on the distance *r* of $\vec {P}$ to the origin and scales approximately as $r^{2}$^21^.

Given an index *j* and the value of $n_j$, an orthogonal strip to $\vec {e}_j$ is built with the tiles produced by taking all the combinations of $\vec {e}_{i}$ with their respective integers $n_{i}$^21^. Using this, we can approximate the integers $n_j$ needed to generate the tiles around $\vec {P}$ as

9 $$\begin{aligned} n_{j} = \Bigg \lfloor \frac{ \vec {P} \cdot \hat{e}_{j}}{d_{A}} \Bigg \rceil , \end{aligned}$$

where $d_{A}$ is the average separation between the bands, $\Bigg \lfloor \cdot \Bigg \rceil$ is the nearest integer and $\hat{e}_{j}$ is the unitary vector in the same direction than $\vec {e}_{j}$.

If the star vectors correspond to the vertices of a regular polygon with *N* sides inscribed in a unit circle, then the distance $d_{A}$ is equal to *N*/2. We only analyzed quasiperiodic systems that satisfy this condition throughout the present work.

Using Eqs. (1) and (9), we can build a set of tiles around the point $\vec {P}$ taking all possible pairs of indices *j*, *k* with their corresponding integers $n^{'}_{j}, n^{'}_{k}$, where $n^{'}_{i} \in [n_{i} - \beta _{i}, n_{i} + \beta _{i}]$ with $\beta _{i} \in \mathbb {Z}^{+} \cup \{0\}$. Here, $\beta$ determines the size of the neighborhood of the quasiperiodic array that we generate and its smallest possible value depends on the symmetry of the system; $\beta _{min} = 1$ for $N<40$ and $\beta _{min} = 0$ for $N \ge 40$.

Since using the GDM we produce each tile with equal probability, our algorithm necessarily produces some tiles that are not connected to each other. As a result, the algorithm produces a set of tiles around $\vec {P}$ that contains that point $\vec {P}$, where the tiles are adjacent to each other (which we will refer to as the main cluster), as well as a set of tiles where the tiles are disconnected from this first set (which we will refer to as the trash tiles). The problem with the trash tiles is that they mostly form clusters of very few or a single tile. Therefore they are not useful for increasing the statistics of nearest neighbor or Voronoi areas distributions.

Eliminating the trash tiles is then a crucial step in our algorithm. The Voronoi cells inside a cluster will have a finite area bounded above by a certain value *A*. In contrast, those tiles at the border of a cluster will have an area greater than *A*. In^21^, this fact is used to generate an iterative algorithm in which tiles are removed from the boundary of the clusters, “evaporating” the small clusters and leaving only the main cluster. A substantial improvement consists of measuring the minimum distance $r_{min}$ between the point $\vec {P}$ and the centroids whose Voronoi cells have an area greater than *A*. By eliminating all the tiles whose centroid are farther than $r_{min}$ from the point $\vec {P}$, we keep only a subset of the tiles of the original main cluster.

Using this improved version of our algorithm, we compute the nearest neighbor distance distribution for a quasiperiodic lattice of rotational symmetry *N* as follows:

1. An arbitrary site $\vec {P}$ is randomly selected within a square region of side $2 \times 10^{6}$ centered at the origin.
2. A main cluster of the quasiperiodic tiling of radius *R* is produced around the site $\vec {P}$. The numerical value of *R* depends on the parameter $\beta$ and on the rotational symmetry *N*, corresponding to the minimum distance $r_{min}$ described in the previous paragraph. The values of the parameters $\alpha _{i}$ (Eq. 1) were set to 0.2 for all values of *i*.
3. We define a circle of radius $R_s = 0.7 R$, centered on $\vec {P}$. The sites of the quasiperiodic lattice that we will analyze to obtain the nearest neighbor distance distribution will be those that are found within this circular region, to avoid boundary tiles.
4. We compute the Voronoi tiling using Fortune algorithm^30^ over the sites of the main cluster. Then, we obtain the first neighbors for each site within the circle of radius $R_s$.
5. We calculate the distance between each one of the sites and its first neighbors, keeping the minimum of these distances in an array.
6. We iterated this procedure until a total of $1 \times 10^{6}$ sites were analyzed.

For the case of the distribution of Voronoi areas, the procedure to follow is the same as the case just exposed, only modifying step 5 for the corresponding calculation of the area of the Voronoi cell associated with each one of the sites within the circle of radius $R_s$ (Supplementary Information).

### Supplementary Information

Supplementary Information 1.

Supplementary Information 2.

## Data Availability

The code of the algorithm used in the present work has been written in JULIA and can be download, as well as the datasets generated during and/or analysed during the current study, from https://github.com/AlanRodrigoMendozaSosa/Quasiperiodic-Tiles.
