# Supplementary material for: Structural studies of local environments in high-symmetry quasicrystals
Source: Sci Rep. 2023 Oct 4;13:16696. doi: 10.1038/s41598-023-42145-7 (PMC10550988; doi:10.1038/s41598-023-42145-7)
Supplement: Supplementary file 1 — Supplementary Information 1. [file 41598_2023_42145_MOESM1_ESM.docx]

Title for the PDF file:

- Supplementary Notes.

Text summary for the PDF file:

- Additional notes to the main text on the invariance of the obtained distributions with respect to isomorphism classes, the statistical sampling performed with representative structures, and the persistence of rotational symmetry of the system in finite regions. Within the document there are four figures with data that support the discussion.
