# Supplementary material for: Structural studies of local environments in high-symmetry quasicrystals
Source: Sci Rep. 2023 Oct 4;13:16696. doi: 10.1038/s41598-023-42145-7 (PMC10550988; doi:10.1038/s41598-023-42145-7)
Supplement: Supplementary file 2 — Supplementary Information 2. [file 41598_2023_42145_MOESM2_ESM.pdf]

Supplementary Information for "Structural studies of local environments in high-symmetry quasicrystals"

Alan Rodrigo Mendoza Sosa[1*], Atahualpa S. Kraemer[1*], Erdal C. Oğuz[2*] and Michael Schmiedeberg[3*]

[1*]Departamento de Física, Facultad de Ciencias, Universidad Nacional Autónoma de México, Ciudad Universitaria, 04510, Mexico City, Mexico.
[2*]CAS Key Laboratory of Soft Matter and Biological Physics, Institute of Physics, Chinese Academy of Sciences, Beijing, 100190, China.
[3*]Institut für Theoretische Physik, Friedrich-Alexander-Universität Erlangen-Nürnberg, 91058 Erlangen, Germany.

*Corresponding author(s). E-mail(s): alanmendoza@ciencias.unam.mx; ata.kraemer@ciencias.unam.mx; ecoguz@iphy.ac.cn; michael.schmiedeberg@fau.de;

Supplementary Notes

Independence of the local isomorphism class

The local statistical properties that we consider, e.g., the nearest-neighbor distance distribution and the Voronoi area distribution are universal in the sense that in the limit of $N \to \infty$ all quasicrystals produced by our method have the same properties, irrespective of the local isomorphism class. We have provided numerical results for one such class in the main text. In Fig. 1 we now show the nearest-neighbor distribution for a quasiperiodic tiling of symmetry $N = 307$ for 10 different local isomorphism classes.

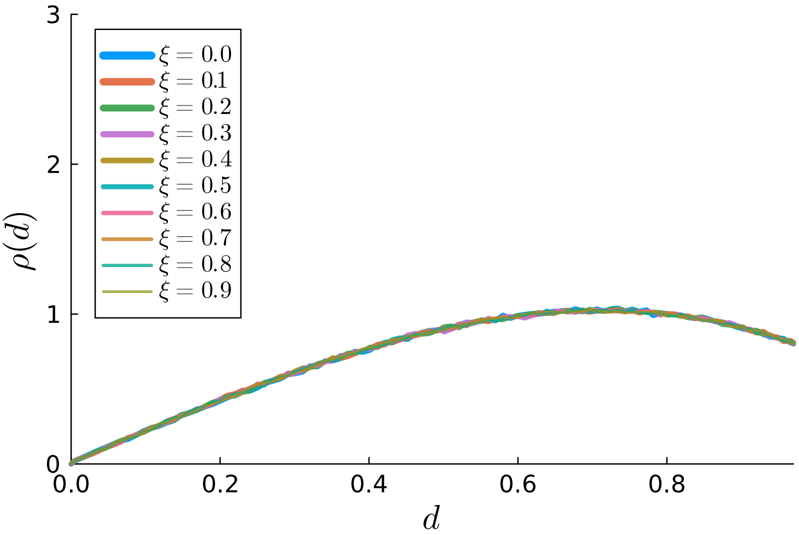

Fig. 1 Nearest-neighbor distribution for different classes of isomorphism of the quasiperiodic tilling with symmetry $N = 307$.

In the generalized dual method explained in the Method section of the main text, there are N *phases* α_i, parameters associated with each of the star vectors $\vec{e}_i$. These parameters can serve to produce a phasonic displacement, but they can be also used to produce different local isomorphism classes. Here a parameter ξ is introduced to distinguish different classes and for the examples shown in Fig. 1 $\alpha_i = \xi/N$ for all i.

Statistical results for different sizes of the sampling area and for different numbers of vertices

To test whether the results shown in Fig. 4 of the main text that have been determined for representative patches correctly describe the distribution of the overall quasicrystal, we determine corresponding results for differently selected patches. Note that patches of the quasicrystal at different distances to the perfect symmetry center might look very different. Figure 2 shows examples of how regions look as a function of the distance to the center of symmetry.

For results in the main text we first choose randomly a point $\vec{P}$ within a square of side $L = 2 \times 10^6$ around the origin. Then we produce a circular region of the quasiperiodic system around $\vec{P}$ in which we calculated the nearest-neighbor distance associated with each of the sites in this region. We continue this procedure until we obtain at least $m = 10^6$ vertices.

Fig. 3 shows the obtained distributions for a quasiperiodic tiling of rotational symmetry $N = 1009$ for different number of vertices as well as different sizes of the square region around the center of symmetry. From left to right, the number of vertices increase, $m = 10^3,\ 10^4,\ 10^5,\ 10^6$. From top to bottom,

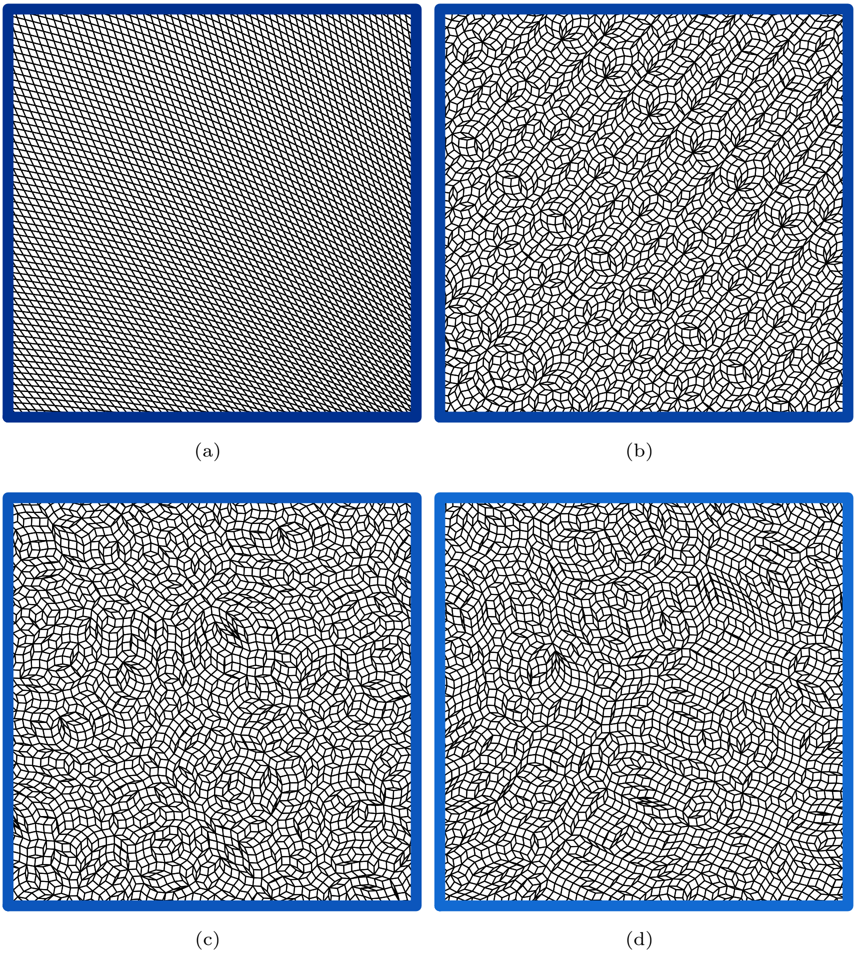

Fig. 2 Examples of neighborhoods at different distances to the center of symmetry for a quasiperiodic tiling with rotational symmetry $N = 1009$. The distances are (a) 10^2, (b) 10^3, (c) 10^4 and (d) 10^5

the side of the square region where we obtained our statistics increases from $L = 2 \times 10^2$ to $L = 2 \times 10^6$ with a factor of 10 between subsequent rows.

As we can see, if the sampling takes place in an area with side length $L = 2 \times 10^4$ or larger then all resulting curves have almost the same form if the number of vertices is 10^6. For $L = 2 \times 10^5$ or larger, the necessary number of vertices to obtain this curve is reduced to 10^5.

In conclusions, the number of vertices and the size of the sampling region that we use in the main text is sufficient to determine the nearest-neighbor distribution of the quasicrystal.

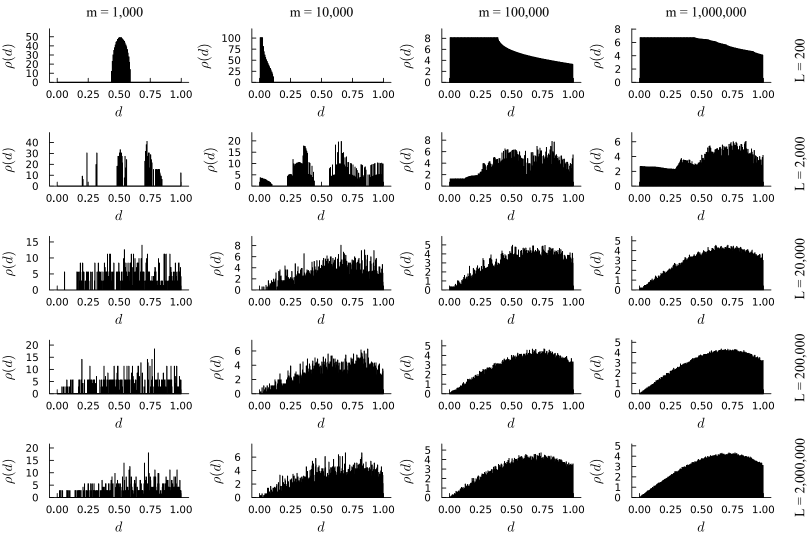

Fig. 3 Nearest-neighbor distance distribution for a quasiperiodic tiling with rotational symmetry $N = 1009$ obtained for different number of vertices (shown in different columns ranging from 10^3 to 10^6 from left to right) and for different sizes of the sampling region (ranging from $L = 2 \times 10^2$ to $L = 2 \times 10^6$ from top to bottom).

Fourier transforms of quasiperiodic configurations

In Fig. 4 we show Fourier transforms of quasiperiodic lattice points for three different symmetries, $N = 7, 11, 13$. Each Fourier transform has been obtained using a single square patch of the corresponding quasicrystal containing $m = 400751, 309953, 405579$ vertices for $N = 7, 11, 13$, respectively. Each of these square patches has been generated around a random site at a distance $d = 10^5$ from the center of symmetry and has subsequently translated to the origin to facilitate analysis. Note that our nearest-neighbor statistics has been achieved with 10^6 or more vertices. Hence, already considerably smaller quasiperiodic patches are able to restore N-fold symmetry as clearly seen by the bright spots in Fig. 4.

Data Availability

The data sets generated and analyzed for this work can be downloaded from https://github.com/AlanRodrigoMendozaSosa/Quasiperiodic-Tiles.

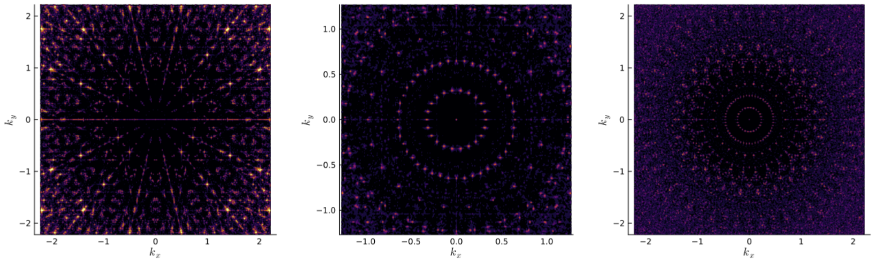

(a) (b) (c)

Fig. 4 Fourier transforms of quasiperiodic configurations with $N = 7$ (a), $N = 11$ (b) and $N = 13$ (c).
